# Increased Measured GFR and Proteinuria in Children with Previous Infection by SARS-CoV-2: Should We Be Concerned?

**DOI:** 10.3390/microorganisms13051008

**Published:** 2025-04-27

**Authors:** Alessia Marcellino, Silvia Bloise, Carmelo Pirone, Giulia Brandino, Antonio Barberi, Emanuela Del Giudice, Vanessa Martucci, Mariateresa Sanseviero, Flavia Ventriglia, Riccardo Lubrano

**Affiliations:** 1 Pediatrics and Neonatology Unit, Maternal-Child Department, Santa Maria Goretti Hospital, Sapienza University of Rome, 04100 Latina, Italy; silvia.bloise1989@gmail.com (S.B.); giulia.brandino@libero.it (G.B.); emanuela.delgiudice@gmail.com (E.D.G.); vany.mart@gmail.com (V.M.); mariateresa.sanseviero@yahoo.it (M.S.); flavia.ventriglia@uniroma1.it (F.V.); 2 Dermatopathic Institute of the Immaculate (IDI-IRCCS), 00167 Rome, Italy; pirone.carmelo@gmail.com; 3 Radiology Unit, Santa Maria Goretti Hospital, Sapienza University of Rome, 04100 Latina, Italy; a.barberi@ausl.latina.it

**Keywords:** COVID-19, SARS-CoV-2, children, kidney, proteinuria

## Abstract

Over the past 3 years, several kidney complications in children with severe involvement by SARS-CoV-2 have been described. However, literature data are still lacking regarding possible kidney injury in children with paucisymptomatic SARS-CoV-2 infection. Thus, we retrospectively evaluated renal function in those patients. Children between 3 and 18 years, without any renal disease, with previous paucisymptomatic SARS-CoV-2 infection from May 2020 to March 2022, were recruited at our post-COVID-19 outpatient clinic. We retrospectively collected: Glomerular filtration rate, Fractional-excretion-of-sodium (FENa), tubular-reabsorption-of-phosphate (TRP), calcium-creatinine-urine ratio (CaU/CrU); proteinuria/m^2^/day and microhematuria by urine cytofluorometry. A total of 148 children were enrolled after a median period of 3 (IQR 6) months after infection. Twenty-six patients (17.6%) had reduced GFR, fifty (33.9%) had hyperfiltration, eleven (7.4%) had abnormal FENa and/or TRP, twenty-two (14.9%) had hypercalciuria, seventy-eight (52.7%) had pathological daily proteinuria. Microhematuria was found in sixteen (10.9%) subjects. Hyperfiltration was more prevalent among males (38.9% vs. 22.4%, *p* = 0.027); CaU/CrU [median 0.08 (IQR 0.09) vs. 0.13 (IQR 0.13) *p* = 0.003] was significantly higher in females. Our data suggest that SARS-CoV-2 could determine, in a significant proportion of children, kidney damage characterized by hyperfiltration, proteinuria, and hematuria, warranting strict follow-up in these patients.

## 1. Introduction

Over the past 3 years, the literature has been enriched with studies and clinical cases regarding SARS-CoV-2 infection in pediatric patients, allowing rapid improvement of knowledge in this area and the application of good medical practice [1]. In addition to the better-known interstitial pneumonia and multi-system inflammatory syndrome in children (MIS-C), several apparatus-specific complications, such as acute heart failure, coronary artery abnormalities, atrioventricular valve insufficiency, encephalopathy, vasculitis, optic neuritis, varicelliform or chilblain-like exanthema, gastroenteritis, and colitis, have been reported in pediatric patients [2].

As regards kidney involvement, a recent review [3] reported how the possible complications have been acute kidney injury (AKI), nephrotic syndrome (both new-onset and relapses), glomerulonephritis, hemolytic-uremic syndrome, vasculitis, collapsing glomerulopathy or rejection in kidney transplants.

According to the most recent literature, it appears that most of the cases involve children with severe systemic involvement by SARS-CoV-2 or individuals in whom the virus may have functioned as a trigger on a genetic predisposition. In a single-center study [4] in Italy, the authors reported kidney involvement in no less than 36% of patients suffering from MIS-C, both as glomerular and tubular damage. Similar results in MIS-C or critically ill children were reported by several authors worldwide [5,6,7,8]. On the other hand, children already suffering from kidney diseases were reported to experience AKI or worsening of their pre-existing condition, as showed by Twichell et al. [9].

However, literature data are lacking regarding possible kidney injury in children with mild SARS-CoV-2 infection. In a retrospective analysis [10], hospitalized children with moderate SARS-CoV-2 infection showed increased levels of proteinuria and urinary markers of AKI than matched healthy controls. No data have been reported regarding kidney involvement in non-hospitalized, previously healthy children.

Therefore, we decided to retrospectively evaluate glomerular and tubular function and proteinuria and hematuria in children with previous infections in order to add knowledge about this unclear involvement.

## 2. Materials and Methods

### 2.1. Patients

All children referring to ASL Latina, with previous SARS-CoV-2 infection from May 2020 to March 2022, were recruited. All parents of these children were subjected, with verbal consent, to telephone interviews designed to assess the date of positivity and negativity on nasopharyngeal swab for SARS-CoV-2, symptomatology, duration of symptoms, and age of the patient.

Inclusion criteria were:

- Diagnosis of SARS-CoV-2 infection made by molecular swab according to regulations at the time of diagnosis; all patients with positive antigenic swab had been confirmed with Polymerase Chain Reaction (PCR) nasopharyngeal swab for SARS-CoV-2. The procedure was performed as follows, according to Fazio et al. instructions [11]: the child was positioned seated, with the head tilted 30–45° upward and toward the operator, in order to have a direct view of the oropharynx. The swab was inserted in the inferomedial angle of the nostril, next to the nasal septum, proceeding along a plane parallel to the hard palate, and with the swab’s end pointed laterally, away from the nasal septum and toward the inferior turbinate. The swab was inserted for at least 9 cm to reach the right target.
- Age at first evaluation between 3 and 18 years;
- Paucisymptomatic infection: rhinitis, fever (>38.5 °C) lasting up to 72 h, cough, asthenia, myalgias, arthralgias, low-grade fever (<38.5 °C lasting up to 6 days);
- Complete evaluation at our post-COVID-19 outpatient clinic of the Unit of Pediatrics of S. Maria Goretti Hospital.
- Evaluation performed at least 2 weeks after a swab resulted negative for SARS-CoV-2.

Exclusion criteria were:

- Personal and family history of renal disease, based on clinical judgment, in particular: proteinuria and/or hematuria, solitary kidney functioning, urinary tract abnormalities, vesicoureteral reflux, glomerulonephritis (both acute and chronic), nephrotic syndrome.
- Personal history of diseases linked to kidney diseases, such as obesity, genetic syndromes (i.e., Down syndrome), diabetes, etc.
- Lack of parenting consent.
- Vaccination for SARS-CoV-2.
- Under or overcollection of urine. All children and parents had been instructed by trained nurses to ensure proper urine collection verbally and through an explanatory illustrated leaflet. When the children delivered their urine containers to the hospital, trained nurses conducted an additional interview aimed at ascertaining that the collection had been conducted properly. An assay on the urinary excretion of creatinine provided an additional approach to assessing the appropriateness of urine collection [12].

### 2.2. Study Protocol

In this cross-sectional study, for every patient, we retrospectively and anonymously collected data regarding:

- Pediatric examination, with blood and urine tests.
- Evaluation of weight, stature, and Body Mass Index (BMI) measures and percentiles according to WHO 2006 growth charts [13]
- Glomerular filtration rate (GFR) as creatinine clearance (GFR_Cr_) [14]:

$${GFR}_{Cr}=\frac{CrU\;\times \;\;\frac{V}{min}}{CrS}\;,$$

where CrU (Randox, Crumlin, County Antrim, Northern Ireland) is the urinary creatinine concentration, CrS (Randox, Crumlin, County Antrim, Northern Ireland) is the serum creatinine concentration and V is the urinary volume collected. We considered as normal the range reported by Heilbron et al. [15].

Renal tubular function. Fractional excretion of sodium (FENa):

$$EFNa=\frac{CrS\times NaU}{CrU\times NaS}\;\times 100$$

where CrU (Randox, Crumlin, County Antrim, Northern Ireland) is the urinary creatinine concentration, CrS (Randox, Crumlin, County Antrim, Northern Ireland) is the serum creatinine concentration, NaU (Thermo Fisher Scientific, Waltham, MA, USA) is the urinary sodium concentration and NaS (Thermo Fisher Scientific, Waltham, MA, USA) is the serum sodium concentration. Tubular reabsorption of phosphate (TRP):

$$RTP=\frac{PU\times CrS}{PS\times CrU}\;\times 100$$

where CrU (Randox, Crumlin, County Antrim, Northern Ireland) is the urinary creatinine concentration, CrS (Randox, Crumlin, County Antrim, Northern Ireland) is the serum creatinine concentration, PU (Atlas Medical GmbH, Blankefelde-Malow, Berlin, Germany) is the urinary phosphate concentration, and PS (Atlas Medical GmbH, Blankefelde-Malow, Berlin, Germany) is the serum phosphate concentration. Calcium–creatinine urine ratio (Thermo Fisher Scientific, Waltham, MA, USA) (CaU/CrU). We considered normal values: FENa < 1%, TRP > 85%, CaU/CrU < 0.8 mg/mg in infants < 6 months, <0.6 mg/mg between 6 and 12 months and <0.2 in older children [16].

- Evaluation of both proteinuria/m^2^/day (Glenbio Ltd., Cork, Ireland) (n.v. < 100 mg/m^2^/day) and presence of microhematuria by urine cytofluorometry (n.v. < 10 Red Blood Cells/μL).
- Evaluation of full abdominal ultrasound.

Every female participated in the full protocol at least 5 days after the end or before the presumed start of their period.

Patients were further divided into three groups according to the duration of infection (from the first positive swab to the first negative one):

- group A: from 1 to 14 days
- group B: from 15 to 26 days
- group C: greater than 27 days

A subsequent division was performed according to the time of evaluation:

- early (assessed within 3 months of infection)
- late (assessed more than 3 months after infection).

Subsequently, we enrolled healthy patients, randomly selected from our kidney outpatient clinic database, in order to find any difference between the two cohorts.

Inclusion criteria were:

- Age-matching with our SARS-CoV-2 cohort
- Sex-matching with our SARS-CoV-2 cohort
- Blood and urine analysis performed before year 2020: Glomerular filtration rate (GFR) as creatinine clearance (GFR_Cr_). We considered as normal the range reported by Heilbron et al. [15], Fractional excretion of sodium (FENa), tubular reabsorption of phosphate (TRP), calcium–creatinine urine ratio (CaU/CrU). We considered normal values: FENa < 1%, TRP > 85%, CaU/CrU < 0.8 mg/mg in infants < 6 months, <0.6 mg/mg between 6 and 12 months and <0.2 in older children [16]. Evaluation of both proteinuria/m^2^/day (n.v. < 100 mg/m^2^/day) and presence of microhematuria by urine cytofluorometry (n.v. < 10 Red Blood Cells/μL).

Exclusion criteria were:

- Personal and family history of renal disease, based on clinical judgment, in particular: proteinuria and/or hematuria, solitary kidney functioning, urinary tract abnormalities, vesicoureteral reflux, glomerulonephritis (both acute and chronic), and nephrotic syndrome.
- Personal history of diseases linked to kidney diseases, such as obesity, genetic syndromes (i.e., Down syndrome), diabetes, etc.

### 2.3. Statistical Analysis

Statistical evaluation was performed using dedicated software: JMP 15.2.1 for MacOs (SAS Institute Inc., Cary, NC, USA), GraphPad 10.4.2 for MacOs (GraphPad Inc., Boston, MA, USA) and SPSS 29.0.0 for MacOs (IBM Statistics, Armonk, NY, USA). Quantitative variables were expressed as mean ± standard deviation (SD) or median and interquartile range (IQR) depending on the sample distribution; qualitative variables were expressed as percentages. Based on the distribution of the data, the comparison of quantitative variables between groups was analyzed using a Student’s *t*-test, Mann–Whitney test, or Wilcoxon test. Comparison between nominal variables was carried out using a chi-square (χ^2^) test or Fisher’s test. The strength of the link between two quantitative variables was measured by Pearson’s r correlation index or Spearman’s r, depending on the distribution of the variables. The relationship between predictors and outcomes was analyzed using simple linear regression models. Multivariate analysis adjusted for main confounders (including age, gender, and BMI) was performed through multiple linear regression.

A two-tailed *p* value of less than 0.05 was considered significant.

## 3. Results

### 3.1. SARS-CoV-2 Population

**Figure 1:**
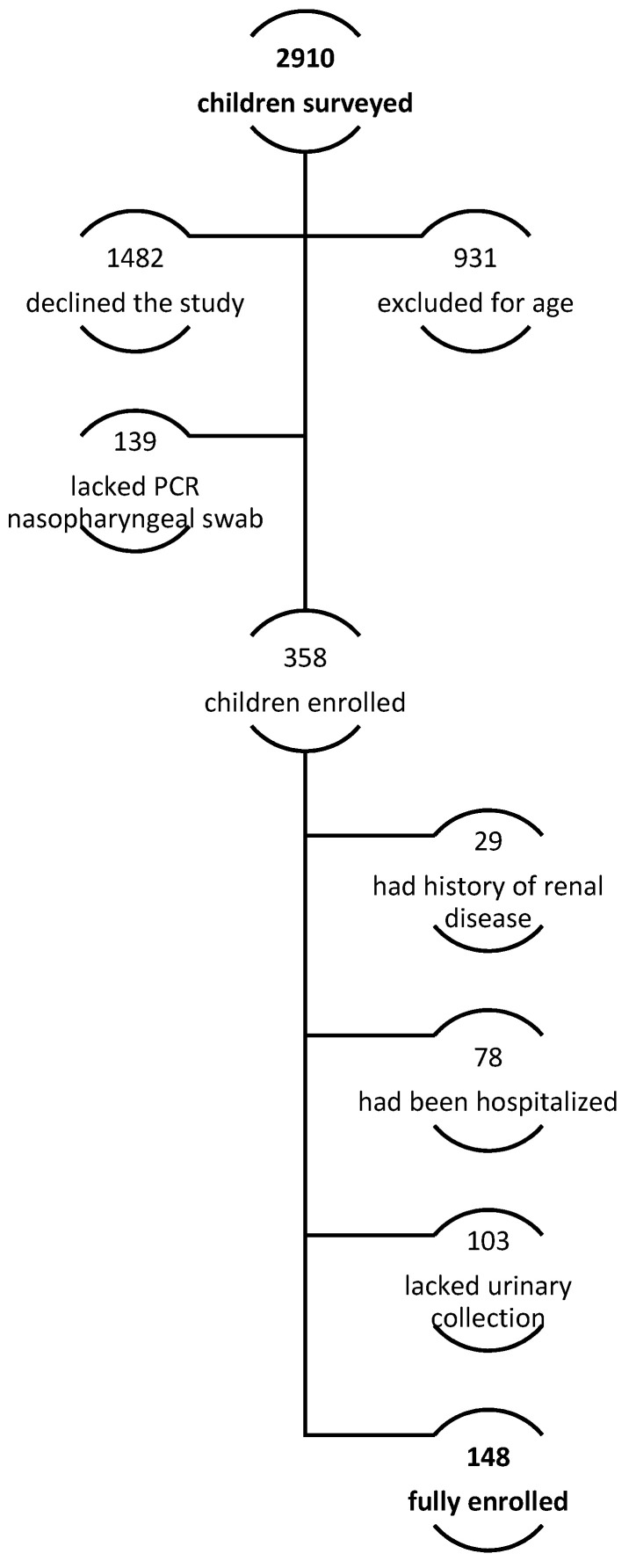
Enrollment protocol.

Of the 2910 parents surveyed, 148 children were enrolled. Indeed, 931 (32.0% of telephonically interviewed subjects) did not meet the inclusion criteria for a lower age. Moreover, most of the patients’ parents, particularly 1482 (50.9%), declined to participate or were not able to participate in the full evaluation. In 139 patients (4.8%), a PCR nasopharyngeal swab had not been performed to confirm SARS-CoV-2 infection, according to national 2022 recommendations; a complete evaluation was not performed in 103 (3.5%) due to incomplete urine collection analysis. Finally, 78 (2.7%) were admitted to the hospital with moderate or severe symptoms (pneumonia, dehydration, Multisystem Inflammatory Syndrome, MIS-C, bacterial co-infection), and 29 (1%) reported a previous history of renal disease (Figure 1). Population characteristics are summarized in Table 1.

Sixty percent of children were male and 39.2% were female. Mean age was 10.1 ± 4.2 years, and median weight, height, and Body Mass Index percentile were in the normal range (respectively, 60 (49.75), 43.5 (52.75), and 71.5 (48.75)).

The latency between SARS-CoV-2 infection and our evaluation was a median of 3 (6) months.

No significant differences based on sex were found in the percentiles of BMI, weight, and height of all the children included in the study.

**Figure 2:**
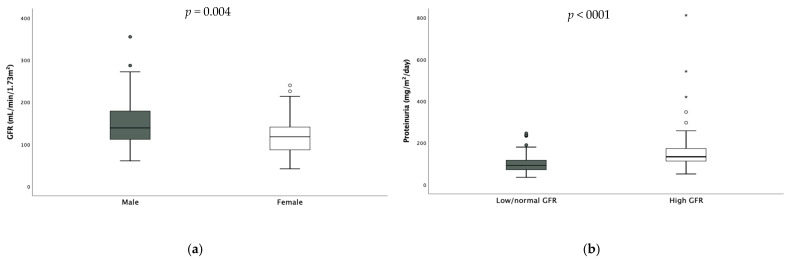
Difference in GFR according to gender (**a**) and difference in daily proteinuria according to GFR (**b**). * extreme outliers.

Median GFR was in the higher range (132 (71.8) mL/min/1.73 m^2^). Twenty-six patients (17.6%) had reduced GFR, while fifty (33.9%) had hyperfiltration. The values of GFR were significantly higher in males [males 139 (67.8) vs. females 118 (55.5) mL/min/1.73 m^2^ *p* = 0.004 (Figure 2)] and hyperfiltration results were more prevalent among them (males 38.9% vs. females 22.4%, *p* = 0.027). Only one patient had undiagnosed monolateral renal hypoplasia shown via abdominal ultrasound.

Also, daily proteinuria was in the higher range (107 (59.75) mg/m^2^/die). Seventy-eight (52.7%) children had pathological proteinuria expressed as mg/m^2^/day—58% of them were hyperfiltrating- and no difference was found according to sex. When considering hyperfiltrating children, daily proteinuria resulted significantly higher in these subjects compared to the others [hyperfiltrating 133.5 (61.7) mg vs. non-hyperfiltrating 92 (45.9) mg, *p* < 0.001, Figure 2].

Among the 78 children with proteinuria, 37 children (48%) had been subsequently reevaluated with diurnal and nocturnal 24 h urine collection in order to exclude orthostatic proteinuria. Only two children had results consistent with this condition.

Median FENa, TRP, calciuria, and urinary red blood cells were normal (0.52 (0.30), 90 (3.75), 0.09 (0.11), 2.5 (4), respectively). Eleven (7.4%) patients had abnormal FENa and/or TRP, and twenty-two (14.9%) had hypercalciuria. Urinalysis did not show any anomalies except microhematuria in sixteen (10.9%) subjects.

**Figure 3:**
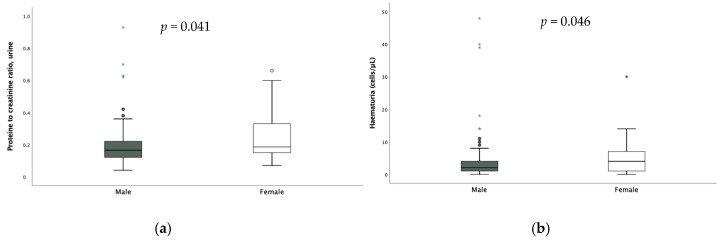
Difference in proteinuria (**a**) and hematuria (**b**) according to sex. * extreme outliers.

No significant difference according to sex was found in the assessment of FENa and TRP; on the other hand, calciuria [0.08 (0.09) vs. 0.13 (0.13) *p* = 0.003], hematuria [2 (3) vs. 4 (6) *p* = 0.046] and proteinuria were significantly higher in females (Figure 3).

No association was found between the presence of hematuria and hypercalciuria.

### 3.2. Analysis According to Time of Evaluation and Duration of Infection

Regarding the duration of viral infection, no statistical significance emerged in the assessment of anthropometric nor of all renal parameters comparing groups A, B, and C.

**Figure 4:**
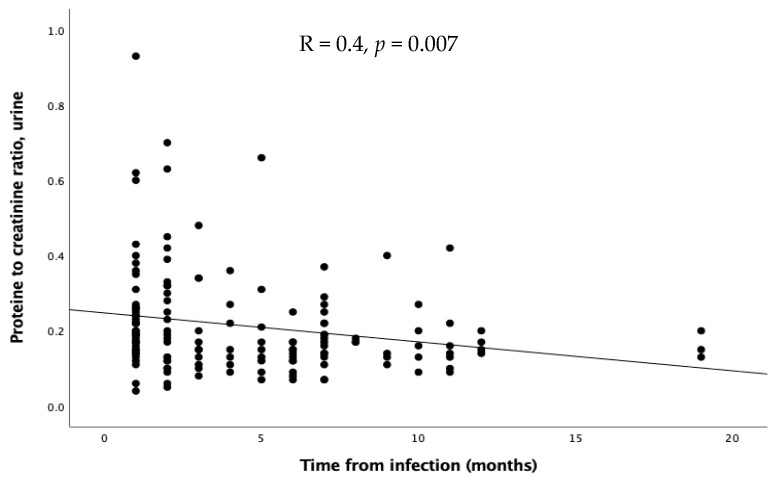
Relation through linear regression between proteinuria and time after infection.

As regards the time of evaluation after the infection, no significant differences emerged in GFR, FENa, and TRP. Proteinuria inversely correlated with time expressed in months (R = 0.4, *p* = 0.0007, Figure 4), while calciuria and urinary red cells showed a similar, but not significant, trend.

In order to confirm our results, we decided to perform a multivariate analysis adjusted for major confounders (including age, gender, and BMI). In particular, male gender, age, and daily proteinuria were confirmed as independent predictors of higher GFR (respectively, *p* = 0.022, *p =* 0.007, *p* < 0.001). Moreover, early evaluation was a predictor of proteinuria, irrespective of gender, age, BMI, and duration of infection (*p* = 0.018). On the other hand, in multivariate analysis, gender did not result as a predictor, and neither did hematuria, proteinuria, or calciuria.

### 3.3. Comparison Between SARS-CoV-2 Patients and Healthy Controls

In our final analysis, we compared renal function between healthy subjects retrospectively enrolled before the COVID-19 pandemic and our SARS-CoV-2 cohort. Our healthy cohort included 51 children: male 58.8%, female 41.2%, mean age was 11.5 ± 3 years, and the BMI percentile was 79.2 (35.3). As regards renal function, GFR was 110 (33.6) mL/min/1.73 m^2^, FENa was 0.61 (0.35)%, TRP was 89 (2.1)%, proteinuria/m^2^/day was 89.5 (20.1) mg, Pr/CrU was 0.15 (0.11), Ca/CrU was 0.10 (0.08) and red blood urinary cells were 3.1 (2).

There was no difference between the two cohorts regarding sex, age, and BMI percentile. GFR was significantly higher in the SARS-CoV-2 cohort (*p* = 0.04), as was proteinuria/m^2^/day (*p* = 0.04). On the other hand, the difference between the two groups in FENa, TRP, Pr/CrU, Ca/CrU, and red blood urinary cells was not statistically significant.

## 4. Discussion

To our knowledge, ours is the first study to extensively evaluate the glomerular and tubular function and the possible presence of renal damage in children with paucisymptomatic SARS-CoV-2 infection. However, we managed to retrospectively study a relatively small sample, mostly due to parents’ refusal to submit to renal assessment during the pandemic; this could be explained by the fear of hospitals during that difficult historical period, the feeling of trying to avoid any excessive stress (i.e., venipuncture) to children, the underestimation of possible and unknown complications in children with paucisymptomatic infection. Even if it could have led to a selection bias, we tried to lessen the risk by consecutively enrolling as many children as we could, excluding all those with possible pre-existing renal conditions and matching them with healthy children studied during the pre-pandemic era.

As regards glomerular function, we decided to investigate it as measured creatinine clearance instead of eGFR. As a matter of fact, even if it is less accurate than another measurement with exogenous markers, it could be a reasonable choice when assessing children without chronic kidney disease, given the lack of radiation exposure. In fact, Adebayo et al. [17] suggested that, since eGFR calculated with Schwartz’s formula was created to evaluate chronic kidney disease (CKD), it might be less accurate when assessing children with hyperfiltration; in addition, most guidelines prescribe the use of eGFR as a screening tool, with measured GFR as confirmation test [18]. On the other hand, it is known that measured creatinine clearance could overestimate GFR by approximately 10% because of tubular secretion [19]. Keeping this in mind, our results still showed a significant prevalence of hyperfiltration, which could be confirmed by follow-up studies and eventually corrected creatinine clearance with cimetidine administration to suppress creatinine tubular secretion.

Males had higher GFR, even when adjusted for confounding factors. This result is not completely new in children. Even if normal GFR values are not sex-adjusted, they depend on height, which is usually different at the same age according to gender. In addition, Pierce et al. proposed new equations with different constants in males and females [20].

Actually, the current literature regarding intrinsic renal damage secondary to SARS-CoV-2 infection—not secondary to severe hypoxia or multi-organ involvement—is composed mainly of clinical cases [21,22,23,24,25,26,27,28,29,30].

In a pilot study by Isoldi et al. [31], the authors evaluated 15 children with previous COVID-19 infection and found hematuria (33%), proteinuria (26%), renal hyperfiltration (33%), and hypofiltration (13%). Our results are similar regarding GFR anomalies but differ in the matter of renal damage. In our study, we found that only 11% of evaluated subjects showed hematuria, but approximately 53% presented not-nephrotic proteinuria, with a much higher prevalence than reported in the pre-COVID-19 era worldwide [32,33,34,35,36,37]. This could be explained by the presence of an acute renal involvement early during the infection (seen as hematuria and proteinuria), with an ameliorating trend after the infection.

A study by Saygili et al. [10] compared children hospitalized due to SARS-CoV-2 associated pneumonia and healthy controls: patients showed significantly higher values of urinary biomarkers for AKI (NGAL-1, KIM-1, IL-18) and albuminuria, even in the absence of proper AKI diagnosis.

In our opinion, as a possible explanation for GFR anomalies [10], SARS-CoV-2 could be implicated in tubular and glomerular damage. It has been postulated that this virus could directly damage the kidneys [38] or dysregulate the renin-angiotensin-aldosterone system and determine cytokine overproduction [39], causing further indirect damage. In addition, glomerular capillaries’ thrombosis had been found in children’s autopsies [40], suggesting that this could be another mechanism for kidney injury. Nonetheless, in children, the role of hypercoagulability is still unclear; for example, retinal vein and artery occlusions seem to be more frequent in the COVID-19 era than before in adults, but surprisingly lower in children less than 9 years old [41].

Renal damage might be one of its most frequent involvements due to the great expression of ACE2 in kidneys [42]. Even if it has been postulated by Cristiani et al. [43] that higher expression of ACE2 in lungs in pediatric patients could inversely correlate with the severity of infection, we have no data regarding pauci-symptomatic kidney involvement in children and the role of ACE2 as a doorway for the virus.

In fact, Puelles et al. [44] found in kidneys from patients who died from COVID-19, SARS-CoV-2 viral load in all kidney compartments examined, especially in glomerular cells, and high levels of RNA for angiotensin-converting enzyme 2 (*ACE2*), transmembrane serine protease 2 (*TMPRSS2*), and cathepsin L (*CTSL*), proteins known to facilitate SARS-CoV-2 kidney infection. In a study by Su et al. [45], Coronavirus-like particles were observed, with principal involvement in the tubular epithelium, and podocytes and immunostaining with SARS-CoV-2 nucleoprotein antibody was positive in tubules. Sharma et al. [46] found similar in-vivo various glomerular and tubular involvements: acute tubular necrosis, thrombotic microangiopathy, crescentic glomerulonephritis, and focal and segmental glomerulosclerosis.

Based on these observations, an early biopsy performed on our patients could have helped clarify the relationship between SARS-CoV-2 infection and kidney damage, but unfortunately, due to the mild symptoms and ethical issues, none of our patients underwent kidney biopsies but were included in a strict follow-up program. In the literature, there are now some data about renal function follow-up. In a study in young adult males [47], it has been proven that patients with pneumonia (considered in our study as a moderate-severe involvement) had higher serum creatinine and blood urea nitrogen (BUN) than asymptomatic patients, with a normalization trend over time.

In addition, Chen et al. [48] reported in adults with previous SARS-CoV-2 infection with no AKI, an increased rate of renal dysfunction when compared to influenza patients both at the 6- and 12-month follow-up, with higher risk in males. This difference persisted also dividing patients according to age into two groups (18–45 and >45 years), while vaccination did not demonstrate any influence on CKD.

The variations we found in the GFR assessment of the children included in the study could be due to a mild renal damage leading to activation of functional reserve and, consequently, hyperfiltration, less seen in our control group. As shown in some CKD models [49,50,51,52] in case of focal (less than 50% of glomeruli) activation of functional reserve, the GFR may remain normal but, in case of diffuse (more than 50% of glomeruli) activation, the GFR may increase, leading to hyperfiltration. Nonetheless, in some cases functional reserve may already be activated at supramaximal level, with reduction of GFR consequently.

In our study, pathological proteinuria could be found both in patients with normal GFR (42% of patients) and hyperfiltration (58% of patients), although higher in the latter; in addition, children with no previous infection had lower proteinuria than children with paucisymptomatic SARS-CoV-2 infection. The proteinuria in children after SARS-CoV-2 infection with normal GFR could be explained by the observation that SARS-CoV-2 could directly damage the podocyte [45] or determine it by cytokine storm [53]. In a case series published by Jung et al. [54], 33% of patients were admitted because of new-onset nephrotic syndrome or glomerulonephritis, even if previously healthy children and with paucisymptomatic SARS-CoV-2 infection.

On the other hand, the highest levels of pathological proteinuria found in our hyperfiltrating children could be explained by a recent review [55] by Chagnac et al. The authors highlighted how hyperfiltration, due to tensile and shear stress, could determine mild proteinuria because of podocyte dysfunction and decreased proximal tubular reabsorption of albumin, as also previously reported by our group [14].

Moreover, in our study, proteinuria inversely correlated with months after infection; thus, it could be possible that SARS-CoV-2 causes some mild damage to the podocyte that tends to ameliorate and resolve with time, except in a small cohort of children, especially with pre-existing renal diseases [56]. Nonetheless, given the cross-sectional nature of our study, this hypothesis needs to be confirmed by more data, possibly with reevaluation of the same children over time. In addition, given the limited access to medical advice during the COVID-19 lockdown [57], it is likely that the prevalence of proteinuria has been significantly higher and underdiagnosed.

As regards SARS-CoV-2 immunization, we decided to focus our study on unvaccinated children. In this field, little is known about possible protection from Acute Kidney injury (AKI) in children; in adults, a study showed no difference in risk of AKI on an immunization basis [58], suggesting that, once the infection has come clean, previous circulating antibodies may not be sufficient to protect the kidneys. On the other hand, a recent review [59] reported some cases (18 in total) of new-onset kidney involvement (IgA nephropathy, tubule interstitial nephritis, and nephrotic syndrome) after SARS-CoV-2 vaccination in children with no prior infection. Among the possible explanations, immune-mediated pathogenesis seems to be the most accountable, even if there is a substantial absence of proper molecular investigation in kidney biopsies.

In our final analysis, we compared our cases with healthy controls. A similar study was conducted by Schmidt-Lauber et al. [60]: COVID-19 patients had lower eGFR, but proteinuria and hematuria were not different between the two cohorts. The difference with our results could be due to the different ages: adults could have less functional reserve and have experienced more kidney-injuring events/diseases, leading to persistent reduction in eGFR and not hyperfiltration.

On the other hand, our study was conducted on unvaccinated children; given the recent review [61] of case series regarding kidney involvement (both relapses and de novo diseases) after SARS-CoV-2 vaccination, it could be useful to conduct a study in the future comparing unvaccinated and vaccinated children.

In conclusion, although the pathogenesis of renal involvement is still unclear, our data suggest that SARS-CoV-2 could result in renal function involvement that should not be underestimated. In fact, as reported in a recent review [62], more and more data are published regarding kidney involvement in SARS-CoV-2 infected children and adults; even if it is probable that this damage heals over time, close follow-up is then necessary because in some cases renal involvement might not improve.

Thus, in the clinical field, it could be advisable to screen for renal function (GFR and proteinuria in particular) in all children with previous SARS-CoV-2 infection, whether asymptomatic or not and, possibly, reevaluate it over time, to early diagnose kidney involvement and suggest the proper lifestyle and pharmacological treatments.

However, further prospective and multicenter studies are warranted to evaluate more precisely the impact of this pandemic infection on kidney and global health.

## Figures and Tables

**Table 1:** Population characteristics (N = 148).

Demographics		
Male	N (%)	90 (60.8%)
Female	N (%)	58 (39.2%)
Age (years)	mean ± SD	10.1 ± 4.2
Weight (percentile)	median (IQR)	60.0 (49.8)
Height (percentile)	median (IQR)	43.5 (52.8)
BMI (percentile)	median (IQR)	71.5 (48.8)
**Renal Function**		
GFR (ml/min/1.73 m^2^)	median (IQR)	132.3 (71.5)
Creatinine (blood, mg/dL)	median (IQR)	0.55 (0.15)
Creatinine (urine, mg/dL)	median (IQR)	92.8 (63.4)
FENa (%)	median (IQR)	0.5 (0.3)
TRP (%)	median (IQR)	90.0 (3.8)
Proteinuria (mg/m^2^/day)	median (IQR)	107.0 (59.8)
PrU/CrU	median (IQR)	0.17 (0.12)
CaU/CrU	median (IQR)	0.09 (0.11)
Hematuria (cells/μL)	median (IQR)	2.5 (4.0)

BMI: Body mass Index; GFR: glomerular filtration rate; FENa: Fractional excretion of sodium; TRP: tubular reabsorption of phosphate; PrU/CrU: protein–creatinine urine ratio; CaU/CrU: calcium–creatinine urine ratio.

## Data Availability

The original contributions presented in this study are included in the article. Further inquiries can be directed to the corresponding author.

## Abbreviations

multi-system inflammatory syndrome in children (MIS-C), acute kidney injury (AKI), Polymerase Chain Reaction (PCR), Body Mass Index (BMI), Glomerular filtration rate (GFR), Fractional excretion of sodium (FENa), tubular reabsorption of phosphate (TRP), calcium-creatinine urine ratio (CaU/CrU).

## References

[1] Lazzerini M., Sforzi I., Trapani S., Biban P., Silvagni D., Villa G., Tibaldi J., Bertacca L., Felici E., Perricone G. (2021). Characteristics and risk factors for SARS-CoV-2 in children tested in the early phase of the pandemic: A cross-sectional study, Italy, 23 February to 24 May 2020. Eurosurveillance.

[2] Seth S., Rashid F., Khera K. (2021). An overview of the COVID-19 complications in paediatric population: A pandemic dilemma. Int. J. Clin. Pract..

[3] Wu H.H.L., Shenoy M., Kalra P.A., Chinnadurai R. (2021). Intrinsic Kidney Pathology Following COVID-19 Infection in Children and Adolescents: A Systematic Review. Children.

[4] Meneghel A., Masenello V., Alfier F., Giampetruzzi S., Sembenini C., Martini G., Tirelli F., Meneghesso D., Zulian F. (2023). Renal Involvement in Multisystem Inflammatory Syndrome in Children: Not Only Acute Kidney Injury. Children.

[5] Basu R.K., Bjornstad E.C., Gist K.M., Starr M., Khandhar P., Chanchlani R., Kelli A. (2022). Acute kidney injury in critically Ill children and young adults with suspected SARS-CoV2 infection. Pediatr. Res..

[6] Stewart D.J., Mudalige N.L., Johnson M., Shroff R., du Pré P., Stojanovic J. (2022). Acute kidney injury in paediatric inflammatory multisystem syndrome temporally associated with SARS-CoV-2 (PIMS-TS) is not associated with progression to chronic kidney disease. Arch. Dis. Child..

[7] Deep A., Upadhyay G., du Pré P., Lillie J., Pan D., Mudalige N., Kanthimathinathan H.K., Johnson M., Riphagen S., Dwarakanathan B. (2020). Acute Kidney Injury in Pediatric Inflammatory Multisystem Syndrome Temporally Associated With Severe Acute Respiratory Syndrome Coronavirus-2 Pandemic: Experience From PICUs Across United Kingdom. Crit. Care Med..

[8] Bjornstad E.C., Krallman K.A., Askenazi D., Zappitelli M., Goldstein S.L., Basu R.K. (2021). Preliminary Assessment of Acute Kidney Injury in Critically Ill Children Associated with SARS-CoV-2 Infection: A Multicenter Cross-Sectional Analysis. Clin. J. Am. Soc. Nephrol..

[9] Twichell S., Ashoor I., Boynton S., Dharnidharka V., Kizilbash S., Erez D.L., Smith J. (2023). COVID-19 disease among children and young adults enrolled in the North American Pediatric Renal Trials and Collaborative Studies registry. Pediatr. Nephrol..

[10] Saygili S., Canpolat N., Cicek R.Y., Agbas A., Yilmaz E.K., Sakalli A.A.K., Aygun D., Akkoc G., Demirbas K.C., Konukoglu D. (2023). Clinical and subclinical acute kidney injury in children with mild-to-moderate COVID-19. Pediatr. Res..

[11] Fazio E., Abousiam M., Caselli A., Accorona R., Nebiaj A., Ermoli I., Erckert B., Calabrese L., Gazzini L. (2020). Proper Procedures for Performing Nasopharyngeal and Oropharyngeal Swabs for COVID-19. ATS Sch..

[12] Lubrano R., Travasso E., Raggi C., Guido G., Masciangelo R., Elli M. (2009). Blood pressure load, proteinuria and renal function in pre-hypertensive children. Pediatr. Nephrol..

[13] WHO Multicentre Growth Reference Study Group (2006). WHO Child Growth Standards: Length/Height-For-Age, Weight-For-Age, Weight-For-Length, Weight-Forheight and Body Mass Index-For-Age: Methods and Development.

[14] Marcellino A., Bloise S., Fraternali R., Pirone C., Brandino G., Testa A., Filippi L., Lubrano R. (2022). Evaluation of Renal Function and Scars in Children With Primary Vesicoureteral Reflux. Urology.

[15] Heilbron D.C., Holliday M.A., Al-Dahwi A., Kogan B.A. (1991). Expressing glomerular filtration rate in children. Pediatr. Nephrol..

[16] Kruse K., Kracht U., Kruse U. (1984). Reference values for urinary calcium excretion and screening for hypercalciuria in children and adolescents. Eur. J. Pediatr..

[17] Adebayo O.C., Nkoy A.B., van den Heuvel L.P., Labarque V., Levtchenko E., Delanaye P., Pottel H. (2023). Glomerular hyperfiltration: Part 2-clinical significance in children. Pediatr. Nephrol..

[18] Levey A.S., Coresh J., Tighiouart H., Greene T., Inker L.A. (2020). Measured and estimated glomerular filtration rate: Current status and future directions. Nat. Rev. Nephrol..

[19] Stevens L.A., Coresh J., Greene T., Levey A.S. (2006). Assessing kidney function--measured and estimated glomerular filtration rate. N. Engl. J. Med..

[20] Pierce C.B., Muñoz A., Ng D.K., Warady B.A., Furth S.L., Schwartz G.J. (2021). Age- and sex-dependent clinical equations to estimate glomerular filtration rates in children and young adults with chronic kidney disease. Kidney Int..

[21] Serafinelli J., Mastrangelo A., Morello W., Cerioni V.F., Salim A., Nebuloni M., Montini G. (2021). Kidney involvement and histological findings in two pediatric COVID-19 patients. Pediatr. Nephrol..

[22] Stewart D.J., Hartley J.C., Johnson M., Marks S.D., du Pré P., Stojanovic J. (2020). Renal dysfunction in hospitalised children with COVID-19. Lancet Child Adolesc. Health.

[23] Alvarado A., Franceschi G., Resplandor E., Sumba J., Orta N. (2021). COVID-19 associated with onset nephrotic syndrome in a pediatric patient: Coincidence or related conditions?. Pediatr. Nephrol..

[24] Shah S.A., Carter H.P. (2020). New-Onset Nephrotic Syndrome in a Child Associated With COVID-19 Infection. Front. Pediatr..

[25] Morreale A., Casciana M.L. (2022). Onset of nephrotic syndrome concomitant to SARS-CoV-2 infection in a 3-year-old child. Pediatr. Nephrol..

[26] Morgan K.M., Imani P.D. (2021). Case report: A 5-year-old with new onset nephrotic syndrome in the setting of COVID-19 infection. BMC Nephrol..

[27] Basalely A., Brathwaite K., Duong M.D., Liu D., Mazo A., Xie Y., Del Rio M., Goilav B., Hayde N., Kaskel F.J. (2021). COVID-19 in Children With Kidney Disease: A Report of 2 Cases. Kidney Med..

[28] Enya T., Morimoto Y., Oshima R., Miyazaki K., Miyazawa T., Okada M., Sugimoto K. (2021). Nephrotic syndrome relapse in a boy with COVID-19. CEN Case Rep..

[29] Basiratnia M., Derakhshan D., Yeganeh B.S., Derakhshan A. (2021). Acute necrotizing glomerulonephritis associated with COVID-19 infection: Report of two pediatric cases. Pediatr. Nephrol..

[30] Fireizen Y., Shahriary C., Imperial M.E., Randhawa I., Nianiaris N., Ovunc B. (2021). Pediatric P-ANCA vasculitis following COVID-19. Pediatr. Pulmonol..

[31] Isoldi S., Mallardo S., Marcellino A., Bloise S., Dilillo A., Iorfida D., Testa A., Del Giudice E., Martucci V., Sanseviero M. (2021). The comprehensive clinic, laboratory, and instrumental evaluation of children with COVID-19, A 6-months prospective study. J. Med. Virol..

[32] Dike A.I., Okechukwu A.A., Ocheke I., Airede K.I. (2021). Asymptomatic Proteinuria and Haematuria in Healthy Public Primary School Children in Abuja, Nigeria. West Afr. J. Med..

[33] Chen M.-C., Wang J.-H., Chu C.-H., Cheng C.-F. (2018). Differential prevalence of hematuria and proteinuria with socio-demographic factors among school children in Hualien, Taiwan. Pediatr. Neonatol..

[34] Jafar T.H., Chaturvedi N., Hatcher J., Khan I., Rabbani A., Khan A.Q., Portman R., Schmid C.H., Levey A.S. (2005). Proteinuria in South Asian children: Prevalence and determinants. Pediatr. Nephrol..

[35] Hothan K.A., Alasmari B.A., Alkhelaiwi O.K., Althagafi K.M., Alkhaldi A.A., Alfityani A.K., Aladawi M.M., Sharief S.N., El Desoky S., Kari J.A. (2016). Prevalence of hypertension, obesity, hematuria and proteinuria amongst healthy adolescents living in Western Saudi Arabia. Saudi Med. J..

[36] Banerjee M., Roy D., Lingeswaran M., Tomo S., Mittal A., Varma P.P. (2022). Urinary Screening in Asymptomatic Indian Children: A Cross Sectional Epidemiological Study. EJIFCC.

[37] Larkins N., Teixeira-Pinto A., Craig J. (2017). The population-based prevalence of albuminuria in children. Pediatr. Nephrol..

[38] Diao B., Wang C., Wang R., Feng Z., Zhang J., Yang H., Tan Y., Wang H., Wang C., Liu L. (2021). Human kidney is a target for novel severe acute respiratory syndrome coronavirus 2 infection. Nat. Commun..

[39] Martinez-Rojas M.A., Vega-Vega O., Bobadilla N.A. (2020). Is the kidney a target of SARS-CoV-2?. Am. J. Physiol. Renal Physiol..

[40] Khairwa A., Jat K.R. (2022). Autopsy findings of COVID-19 in children: A systematic review and meta-analysis. Forensic Sci. Med. Pathol..

[41] Park H.S., Kim S., Lee C.S., Byeon S.H., Kim S.S., Lee S.W., Kim Y.J. (2023). Retinal vascular occlusion risks during the COVID-19 pandemic and after SARS-CoV-2 infection. Sci. Rep..

[42] Hamming I., Timens W., Bulthuis M.L.C., Lely A.T., Navis G.J., van Goor H. (2004). Tissue distribution of ACE2 protein, the functional receptor for SARS coronavirus. A first step in understanding SARS pathogenesis. J. Pathol..

[43] Cristiani L., Mancino E., Matera L., Nenna R., Pierangeli A., Scagnolari C., Midulla F. (2020). Will children reveal their secret? The coronavirus dilemma. Eur. Respir. J..

[44] Puelles V.G., Lütgehetmann M., Lindenmeyer M.T., Sperhake J.P., Wong M.N., Allweiss L., Chilla S., Heinemann A., Wanner N., Liu S. (2020). Multiorgan and Renal Tropism of SARS-CoV-2. N. Engl. J. Med..

[45] Su H., Yang M., Wan C., Yi L.-X., Tang F., Zhu H.-Y., Yi F., Yang H.C., Fogo A.B., Nie X. (2020). Renal histopathological analysis of 26 postmortem findings of patients with COVID-19 in China. Kidney Int..

[46] Sharma P., Uppal N.N., Wanchoo R., Shah H.H., Yang Y., Parikh R., Khanin Y., Madireddy V., Larsen C.P., Jhaveri K.D. (2020). COVID-19-Associated Kidney Injury: A Case Series of Kidney Biopsy Findings. J. Am. Soc. Nephrol..

[47] Al Rumaihi K., Khalafalla K., Arafa M., Nair A., Al Bishawi A., Fino A., Sirtaj F., Ella M.K., ElBardisi H., Khattab M.A. (2023). COVID-19 and renal involvement: A prospective cohort study assessing the impact of mild SARS-CoV-2 infection on the kidney function of young healthy males. Int. Urol. Nephrol..

[48] Chen I.-W., Chang L.-C., Ho C.-N., Wu J.-Y., Tsai Y.-W., Lin C.-M., Chang Y.J., Hung K.C. (2025). Association between COVID-19 and the development of chronic kidney disease in patients without initial acute kidney injury. Sci. Rep..

[49] Brenner B.M. (1985). Nephron adaptation to renal injury or ablation. Am. J. Physiol..

[50] Anderson S., Brenner B.M. (1986). The role of intraglomerular pressure in the initiation and progression of renal disease. J. Hypertens. Suppl..

[51] Neuringer J.R., Brenner B.M. (1993). Hemodynamic theory of progressive renal disease: A 10-year update in brief review. Am. J. Kidney Dis..

[52] Neuringer J.R., Brenner B.M. (1992). Glomerular hypertension: Cause and consequence of renal injury. J. Hypertens. Suppl..

[53] Ye Q., Wang B., Mao J. (2020). The pathogenesis and treatment of the ‘Cytokine Storm’ in COVID-19. J. Infect..

[54] Jung J., Lee J., Lee J.H. (2023). Kidney involvement in children during the SARS-CoV-2 Omicron variant pandemic. BMC Pediatr..

[55] Chagnac A., Zingerman B., Rozen-Zvi B., Herman-Edelstein M. (2019). Consequences of Glomerular Hyperfiltration: The Role of Physical Forces in the Pathogenesis of Chronic Kidney Disease in Diabetes and Obesity. Nephron.

[56] Morello W., Vianello F.A., Proverbio E., Peruzzi L., Pasini A., Montini G. (2022). COVID-19 and idiopathic nephrotic syndrome in children: Systematic review of the literature and recommendations from a highly affected area. Pediatr. Nephrol..

[57] Lubrano R., Villani A., Berrettini S., Caione P., Chiara A., Costantino A., Formigari R., Franzoni E., Gattinara G.C., Giustardi A. (2020). Point of view of the Italians pediatric scientific societies about the pediatric care during the COVID-19 lockdown: What has changed and future prospects for restarting. Ital. J. Pediatr..

[58] Borrego-Moreno J.C., Cárdenas-de Luna M.J., Márquez-Castillo J.C., Reyes-Ruiz J.M., Osuna-Ramos J.F., León-Juárez M., Del Ángel R.M., Rodríguez-Carlos A., Rivas-Santiago B., Farfan-Morales C.N. (2024). Acute Kidney Injury in the Context of COVID-19: An Analysis in Hospitalized Mexican Patients. Infect. Dis. Rep..

[59] Wu H.H.L., Shenoy M., Kalra P.A., Chinnadurai R. (2022). Intrinsic Kidney Pathology in Children and Adolescents Following COVID-19 Vaccination: A Systematic Review. Children.

[60] Schmidt-Lauber C., Hänzelmann S., Schunk S., Petersen E.L., Alabdo A., Lindenmeyer M., Hausmann F., Kuta P., Renné T., Twerenbold R. (2023). Kidney outcome after mild to moderate COVID-19. Nephrol. Dial. Transplant..

[61] Baek H.S., Cho M.H. (2023). Kidney complications associated with COVID-19 infection and vaccination in children and adolescents: A brief review. Clin. Exp. Pediatr..

[62] Rai V. (2023). COVID-19 and Kidney: The Importance of Follow-Up and Long-Term Screening. Life.

